# 74. Clinical and Microbiology Outcomes of Bloodstream Infections (BSI) in Adults Undergoing Allogeneic Hematopoietic Stem Cell Transplant (allo-HCT) in a Randomized, Double-blind, Placebo-controlled Cohort 2 of a Phase 1b Study of SER-155, an Investigational Live Biotherapeutic

**DOI:** 10.1093/ofid/ofaf695.025

**Published:** 2026-01-11

**Authors:** Tessa Andermann, David Fredricks, Bina Tejura, David Lichter, Brooke Hasson, Meghan Chafee, Nathan Hicks, Mary-Jane Lombardo, Christopher Ford, Matt Henn, Dennis M Walling

**Affiliations:** University of North Carolina at Chapel Hill, Chapel Hill, NC; Fred Hutchinson Cancer Research Center; University of Washington, Seattle, WA; Seres Therapeutics, Cambridge, Massachusetts; Seres Therapeutics, Cambridge, Massachusetts; Seres Therapeutics, Cambridge, MA, Cambridge, Massachusetts; Seres Therapeutics, Cambridge, Massachusetts; Seres Therapeutics, Cambridge, Massachusetts; Seres Therapeutics, Inc, Cambridge, Massachusetts; Seres Therapeutics, Inc, Cambridge, Massachusetts; Seres Therapeutics, Inc., Cambridge, MA; Seres Therapeutics, Cambridge, Massachusetts

## Abstract

**Background:**

SER-155 is an investigational, oral live biotherapeutic, comprised of 16 bacterial strains, designed to protect GI mucosal barrier integrity and prevent BSI in patients receiving allo-HCT. FDA granted Breakthrough Therapy Designation to SER-155 based on results of a Phase 1b trial in adults undergoing allo-HCT that showed SER-155 was well-tolerated and associated with significantly lower incidence of BSI compared with placebo (10% vs 43%, respectively; Odds Ratio 0.15 [95% CI, 0.01-1.13]; p=0.0423). Concentrations of fecal albumin, a biomarker of impaired GI barrier integrity, were significantly higher (p=0.04; *post hoc*) pre-HCT in participants with BSI post-HCT, consistent with the GI translocation hypothesis of BSI pathogenesis. Here, we report new *post hoc* summaries of clinical and microbiology outcomes in participants with BSI.

**Figure:**
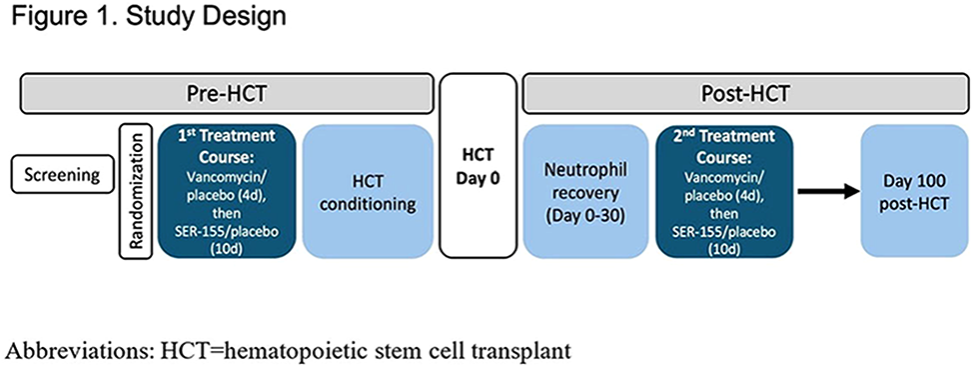

**Figure:**
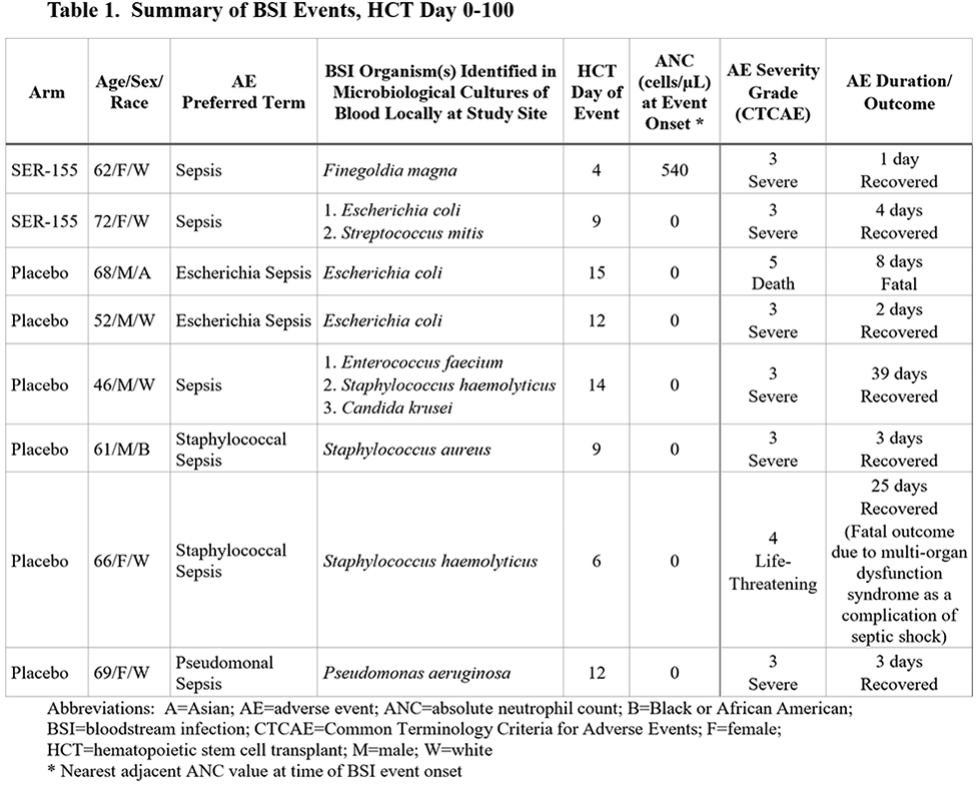

**Methods:**

Adult patients were randomized 1:1 to receive vancomycin/SER-155 or placebo/placebo administered ppre-HCT and post-neutrophil recovery (Figure 1). BSI within HCT Day 0-100 were summarized by treatment arm, causative organism, clinical outcome, antibacterial prophylaxis (AP), and antimicrobial resistance (AMR) test results.

**Figure:**
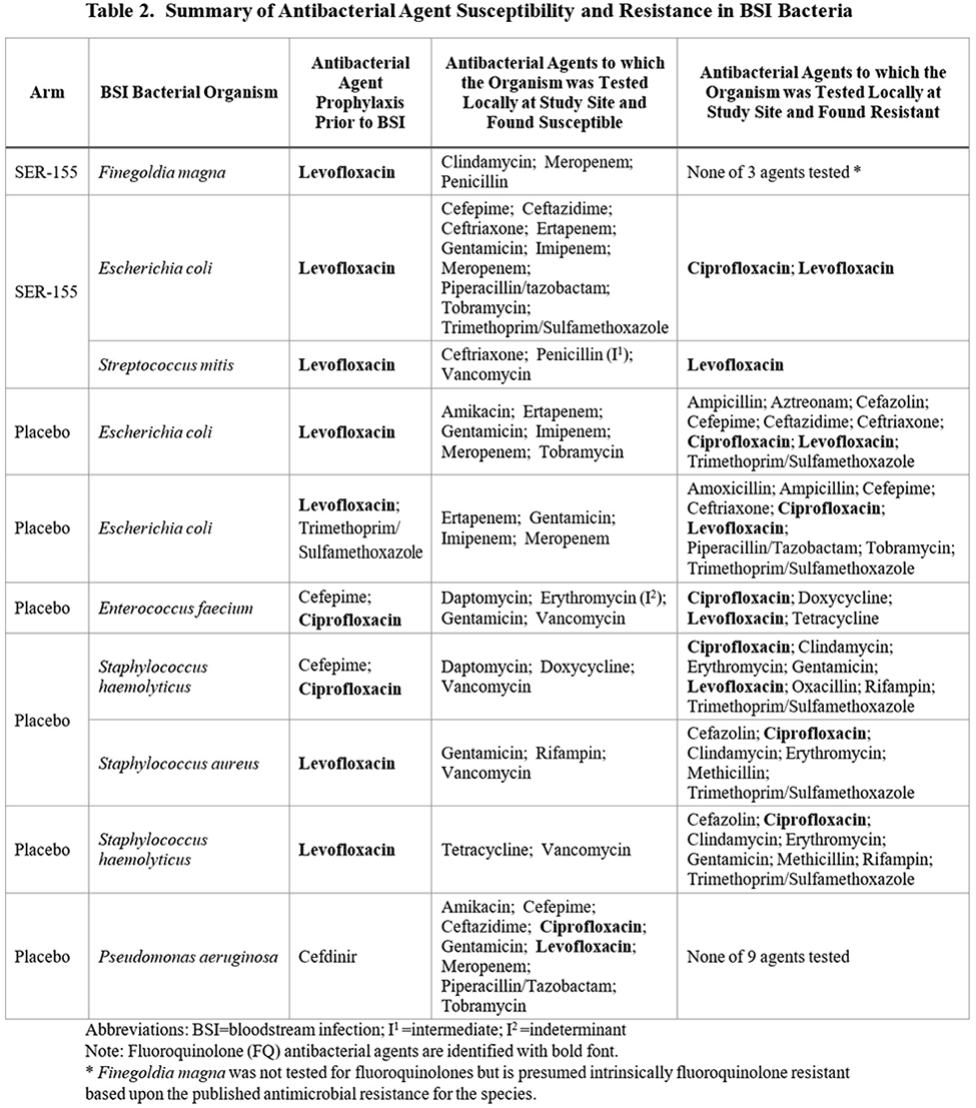

**Results:**

34 participants (SER-155 = 20; placebo = 14) were treated and received allo-HCT. 2 (10%) treated with SER-155 and 6 (43%) treated with placebo had BSI caused by ≥1 bacterial or fungal organism. 2 BSI were polymicrobial. Organisms were consistent with typical BSI pathogens after allo-HCT; no SER-155 species were identified in any BSI. All BSI occurred between HCT Days 4 and 15, before neutrophil recovery. All BSI were treated with antimicrobial agents as per local standard of care. 2 of 6 placebo participants had fatal outcomes (Table 1). All participants with BSI received AP, with all but 1 receiving a fluoroquinolone (FQ). All FQ resistant BSI bacteria were in participants receiving FQ AP. 5 of 6 placebo, but 0 of 2 SER-155, participants had BSI bacteria with multidrug AMR (MDR) (Table 2).

**Conclusion:**

BSI incidence was lower with SER-155 than placebo, and BSI occurred despite AP. BSI bacteria exhibited high prevalence FQ resistance and other AMR, consistent with published reports of BSI bacteria in allo-HCT. Two placebo participants with MDR BSI bacteria had fatal outcomes.

**Disclosures:**

Tessa Andermann, MD, MPH, Seres Therapeutics: Advisor/Consultant

